# Odd-Numbered Agaro-Oligosaccharides Produced by α-Neoagaro-Oligosaccharide Hydrolase Exert Antioxidant Activity in Human Dermal Fibroblasts

**DOI:** 10.3390/md22110495

**Published:** 2024-11-03

**Authors:** Eunyoung Jo, Navindu Dinara Gajanayaka, Minthari Sakethanika Bandara, Svini Dileepa Marasinghe, Gun-Hoo Park, Su-Jin Lee, Chulhong Oh, Youngdeuk Lee

**Affiliations:** 1 Korea Institute of Ocean Science & Technology (KIOST), Jeju-si 63349, Republic of Korea; jey8574@kiost.ac.kr (E.J.); navindu@kiost.ac.kr (N.D.G.); minthari@kiost.ac.kr (M.S.B.); svini91@kiost.ac.kr (S.D.M.); gunhoopark@kiost.ac.kr (G.-H.P.); rbcwbc@kiost.ac.kr (S.-J.L.); 2 Department of Marine Biotechnology, KIOST School, Korea National University of Science and Technology, Daejeon 34113, Republic of Korea

**Keywords:** agarase, agar oligosaccharide, antioxidant, human dermal fibroblasts

## Abstract

Agarases produce agar oligosaccharides with various structures exhibiting diverse physiological activities. α-Neoagaro-oligosaccharide hydrolase (α-NAOSH) specifically cleaves even-numbered neoagaro-oligosaccharides, producing 3,6-anhydro-l-galactose (l-AHG) and odd-numbered agaro-oligosaccharides (OAOSs). In this study, α-NAOSH from the agar-degrading marine bacterium *Gilvimarinus agarilyticus* JEA5 (Gaa117) was purified and characterized using an *E. coli* expression system to produce OAOSs and determine their bioactivity. Recombinant Gaa117 (rGaa117) showed maximum activity at pH 6.0 and 35 °C. rGaa117 retained >80% of its initial activity after 120 min at 30 °C. The activity was enhanced in the presence of Mn^2+^. *K_m_*, *V_max_*, and *K_cat_*/*K_m_* values of the enzyme were 22.64 mM, 246.3 U/mg, and 15 s^−1^/mM, respectively. rGaa117 hydrolyzed neoagarobiose, neoagarotetraose, and neoagarohexaose, producing OAOSs that commonly contained l-AHG. Neoagarobiose and neoagarotetraose mixtures, designated NAO24, and mixtures of l-AHG and agarotriose, designated AO13, were obtained using recombinant rGaa16B (β-agarase) and rGaa117, respectively, and their antioxidant activities were compared. AO13 showed higher hydrogen peroxide-scavenging activity than NAO24 in human dermal fibroblasts in vitro because of structural differences: AOSs have d-galactose at the non-reducing end, whereas NAOSs have l-AHG. In conclusion, OAOSs exhibited high ROS-scavenging activity in H_2_O_2_-induced human dermal fibroblasts. They may be applicable in cosmetics and pharmaceuticals for prevention of skin aging.

## 1. Introduction

Agarose, a polysaccharide derived from agar, is composed of repeating units of d-galactose (d-Gal) and 3,6-anhydro-l-galactose (l-AHG) bonded via alternating α-1,3 and β-1,4-glycosidic linkages [1]. Through hydrolysis, agarose is converted into low-molecular-weight sugars known as agar oligosaccharides, which can be classified into two main types: agaro-oligosaccharides (AOSs) and neoagaro-oligosaccharides (NAOSs). AOSs are characterized by having d-Gal at the non-reducing end, whereas NAOSs have l-AHG at the non-reducing end [2,3]. Agar oligosaccharides can be obtained through chemical or enzymatic methods. Chemical methods involve the use of acids such as citric acid, hydrochloric acid, or solid acids and offer the benefits of having a low cost and a high production rate. However, these methods present important challenges, including environmental pollution and structural instability [3,4]. Considering these factors, enzymatic methods using agarases offer even more significant advantages. Agarases are classified into α-agarases and β-agarases according to their cleavage mode [5]. α-Agarases hydrolyze α-1,3 glycosidic linkages, producing even-numbered AOSs (EAOSs), which are further degraded by β-galactosidase, which acts on the β-1,4-glycosidic linkage at the non-reducing end of EAOSs, producing galactose and odd-numbered NAOSs (ONAOSs) [6]. In contrast, β-agarases produce even-numbered NAOSs (ENAOSs) by cleaving β-1,4-glycosidic linkages. Subsequently, α-neoagaro-oligosaccharide hydrolase (α-NAOSH) can cleave the α-1,3 glycosidic linkage at the non-reducing end of ENAOSs, producing odd-numbered agaro-oligosaccharides (OAOSs) and l-AHG. Carbohydrate-activating enzymes are grouped based on their amino-acid sequence similarity [7]. Glycoside hydrolases (GHs) form the largest class and cleave glycosidic bonds. α-Agarases belong to distinct GH families, such as GH96 and GH117, and β-agarases belong to GH16, GH39, GH50, GH86, and GH118 [8,9].

Agar oligosaccharides have diverse structures and degrees of polymerization (DPs), leading to differences in their physiological activities [3]. Studies have demonstrated that AOSs exert beneficial effects, including anti-inflammatory effects by inhibiting nitric oxide production in RAW264.7 cells [10], antitumor properties by mitigating gut dysbiosis in mice [11], and prebiotic activity by promoting the growth of probiotic bacteria in mice [12]. ENAOSs have shown antidiabetic activity by inhibiting α-glucosidase activity in vitro and whitening activity by lowering melanin contents in Melana-A cells [13]. Notably, most AOSs used in previous studies were produced using the chemical method, and most ENAOSs were produced using β-agarase. From an industrial perspective, using an enzymatic method to produce agar oligosaccharides offers numerous advantages. However, research on the activities of other agar oligosaccharides generated by agarases, besides ENAOSs, is insufficient. Therefore, it is necessary to study the production of agar oligosaccharides with more diverse structures by combining various agarases.

Cells produce reactive oxygen species (ROS), including the superoxide anion (·O2^−^), hydroxyl radical (·OH), hydroxyl ion (OH^−^), and hydrogen peroxide (H_2_O_2_), during oxygen metabolism. Excess ROS induce oxidative stress, which is implicated in aging due to DNA, protein, and lipid damage. Antioxidants such as ascorbic acid, vitamin E, and polyphenols have been reported to reduce ROS levels and oxidative stress [14,15,16]. Additionally, AOSs obtained through acid hydrolysis have shown a hepatoprotective effect in H_2_O_2_-induced cells [17]. Based on these findings, agar oligosaccharides have potential as antioxidants for preventing diseases caused by oxidative stress. However, the effects of agar oligosaccharides in human dermal fibroblasts (HDFs) have not been studied. Therefore, we assessed the antioxidant activities of ENAOSs and OAOSs produced by agarase in HDFs.

In this study, an α-NAOSH (termed Gaa117) from the agar-degrading marine bacterium *Gilvimarinus agarilyticus* JEA5 was overexpressed and biochemically characterized. To compare the activities of agar oligosaccharides in terms of their structural differences, a mixture of ENAOSs, including neoagarobiose and neoagarotetraose, was produced using a recombinant β-agarase and then treated with recombinant Gaa117 to obtain a mixture of OAOSs, including l-AHG and agarotriose. Subsequently, their antioxidant activities were investigated in HDFs.

## 2. Results

### 2.1. Sequence Analysis and Cloning of rGaa117

**Figure 1:**
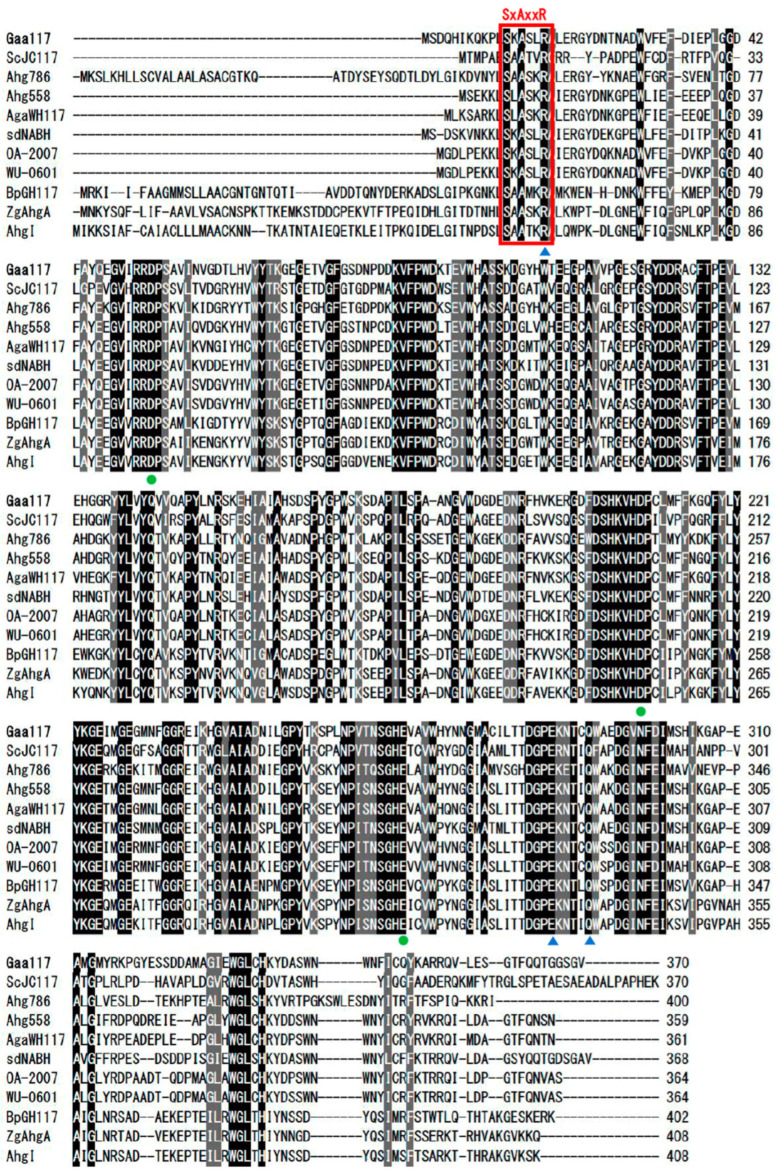
Amino-acid alignment of Gaa117 and other α-NAOSHs. Circles (●) denote catalytic residues and triangles (▲) indicate residues involved in dimer formation. GenBank accession numbers are CAB61805 for ScJC117 from *Streptomyces coelicolor* A3(2), WP_017446786.1 for Ahg786 from *Gayadomonas joobiniege*, WP_017446558.1 for Ahg558 from *Gayadomonas joobiniege*, WP_055733245.1 for AgaWH117 from *Agarivorans gilvus*, WP_011469134.1 for SdNABH from *Saccharophagus degradans*, BAQ55620.2 for OA-2007 from *Cellvibrio* sp., BAV72142.1 for WU-0601 from *Cellvibrio* sp., WP_007560917 for BpGH117 from *Bacteroides plebeius*, WP_013995985.1 for ZgAhgA from *Zobellia galactanivorans*, and WP_077400332.1 for AhgI from *Cellulophaga omnivescoria*.

The sequence of Gaa117 has been deposited in NCBI under accession number PQ417409. The open reading frame of Gaa117 consists of 1113 bp nucleotides that encode a 370-amino-acid protein. The predicted molecular weight of Gaa117 is 41.4 kDa, and its isoelectric point is 5.11. Protein sequence alignment revealed that, like other GH117 family members, Gaa117 harbors conserved regions. The SxAxxR motif and conserved Arg-17, Glu-286, and Gln-291 residues imply that Gaa117 forms a dimer. Moreover, the Asp-53, Asp-208, and Glu-266 residues, which play a role in the catalytic sites, are conserved (Figure 1).

**Figure 2:**
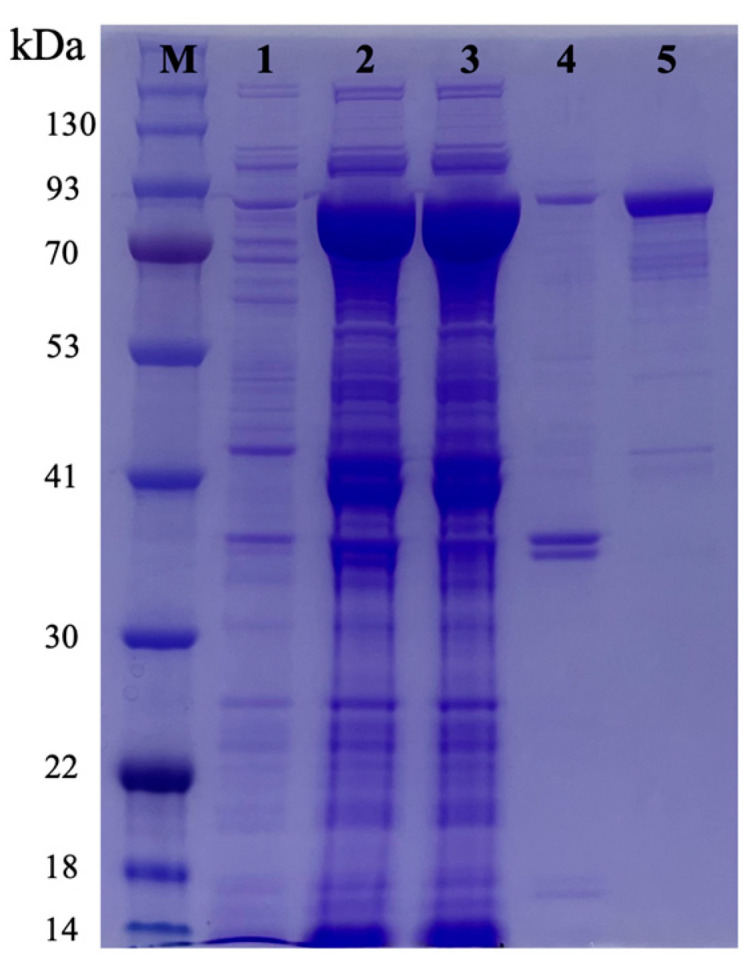
SDS-PAGE analysis of rGaa117. Lanes: M, molecular mass marker; 1, total cell protein before induction; 2, total cell protein after IPTG induction; 3, total cellular soluble extract after induction; 4, total cellular insoluble extract after induction; 5, purified rGaa117.

Recombinant Gaa117 (rGaa117) was overexpressed in *E. coli* (BL21) and purified, and sodium dodecyl sulfate polyacrylamide gel electrophoresis (SDS-PAGE) showed that rGaa117 was obtained mostly in the soluble form. The molecular weight of rGaa117 purified from the soluble fraction was approximately 84 kDa, consistent with the predicted molecular weight (maltose-binding protein 42.5 kDa + rGaa117 41.4 kDa) (Figure 2).

### 2.2. Enzymatic Properties of rGaa117

**Figure 3:**
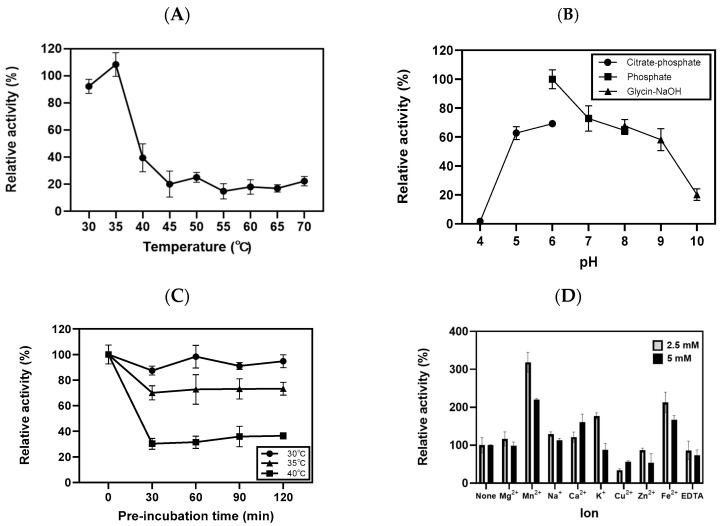
Biochemical properties of rGaa117 activity toward neoagarobiose. Effects of temperature (**A**), pH (**B**), thermostability (**C**), and metal ions and EDTA (**D**) on rGaa117 activity.

The optimal enzyme activity of rGaa117 was analyzed using the dinitrosalicylic acid method. As shown in Figure 3A, maximum rGaa117 activity was observed at 35 °C, and activity sharply declined above the optimum temperature. Enzyme activity was investigated in a pH range of 4.0–10.0, which revealed that the highest activity was achieved at pH 6.0 in phosphate buffer. More than 60% of the activity was maintained in the pH range of 6.0–8.0 (Figure 3B). Next, the residual activity of rGaa117 after pre-incubation was determined. rGaa117 retained more than 80% and 60% of its initial activity after a 120 min pre-incubation at 30 °C and 35 °C, respectively. However, after pre-incubation at 40 °C for 120 min, the enzyme activity was less than 40% of the initial activity (Figure 3C). We next investigated the effects of various reagents on rGaa117 activity. The activity was inhibited in the presence of CuSO_4_, ZnSO_4_, or ethylenediamine tetraacetic acid (EDTA), whereas it was enhanced in the presence of MnCl_2_, NaCl, CaCl_2,_ or FeSO_4_. Further, rGaa117 activity was increased in the presence of 2.5 mM KCl, but decreased in the presence of 5 mM KCl. It should be noted that MnCl_2_ (2.5 mM) enhanced rGaa117 activity by 3.19-fold compared with the control (Figure 3D). The *K_m_*, *V_max_*, and *K_cat_* values of rGaa117 were 22.64 mM, 246.3 U/mg, and 340.7 s^−1^, respectively.

### 2.3. Hydrolyzation Products of rGaa117 and Production of Mixtures NAO24 and AO13

**Figure 4:**
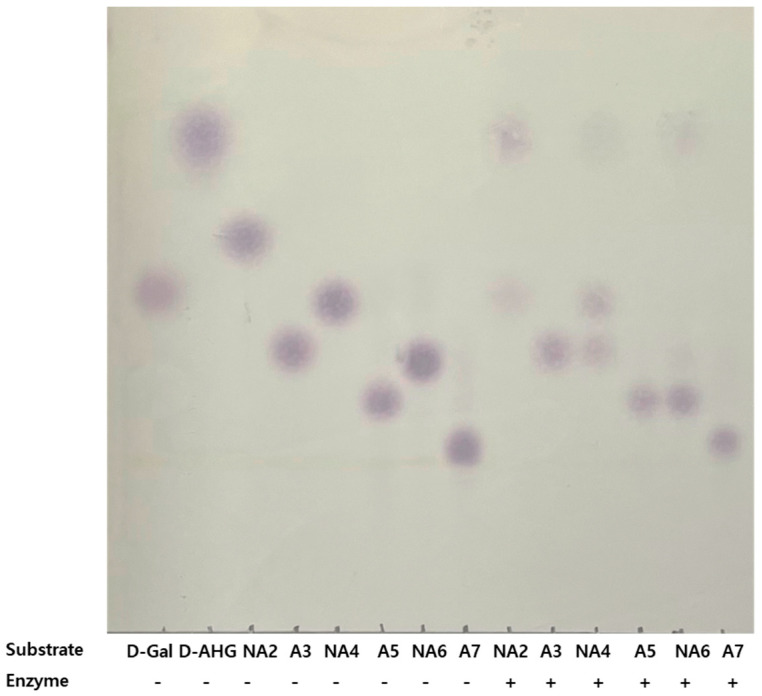
TLC of hydrolysis products by rGaa117 on ENAOSs and OAOSs. d-Gal, d-galactose; d-AHG, 3,6-anhydro-d-galactose; NA2, neoagarobiose; A3, agarotriose; NA4, neoagarotetraose; A5, agaropentaose; NA6, neoagarohexaose; A7, agaroheptaose.

**Figure 5:**
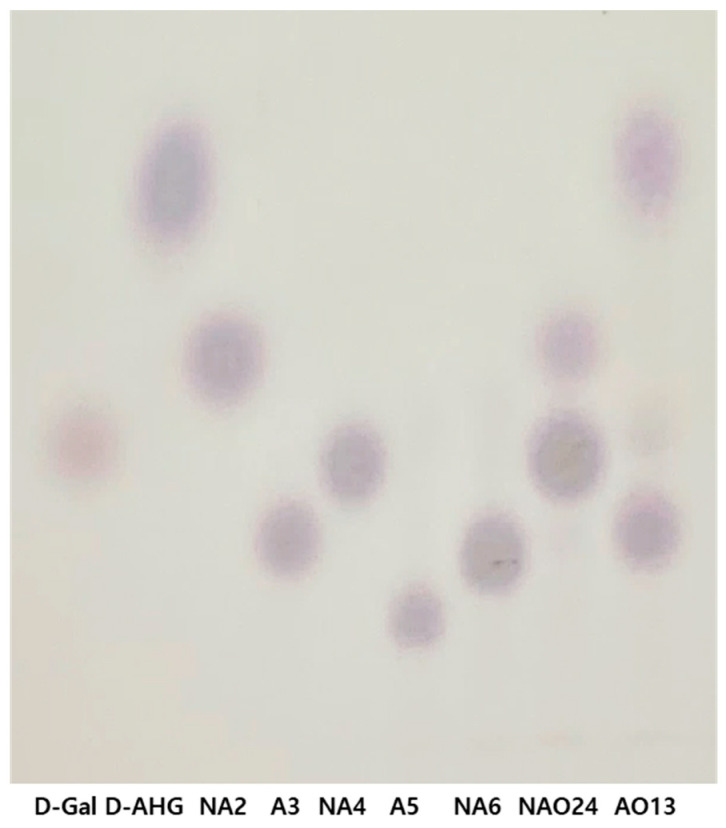
TLC of NAO24 and AO13. NAO24:agarose hydrolyzed with β-agarase; AO13:NAO24 hydrolyzed with rGaa117.

The hydrolyzation products of several rGaa117 substrates were separated on a thin-layer-chromatography (TLC) plate. rGaa117 hydrolyzed neoagarobiose (NA2), neoagarotetraose (NA4), and neoagarohexaose (NA6) into d-Gal, agarotriose (A3), and agaropentaose (A5), which all contain l-AHG. The extent of hydrolysis varied depending on the substrate. NA2 and NA6 were almost completely hydrolyzed, whereas NA4 was less hydrolyzed. Gaa117 did not hydrolyze A3, A5, or agaroheptaose (A7) (Figure 4). These results revealed that Gaa117 is an α-NAOSH that hydrolyzes ENAOSs into l-AHG and OAOSs. The production of mixtures composed of NA2 and NA4 (designated NAO24) by Gaa16B and of l-AHG, A3, and a small amount of d-Gal (designated AO13) by rGaa117 was confirmed using TLC (Figure 5).

### 2.4. H_2_O_2_-Scavenging Activities of NAO24, AO13, and d-Gal

**Figure 6:**
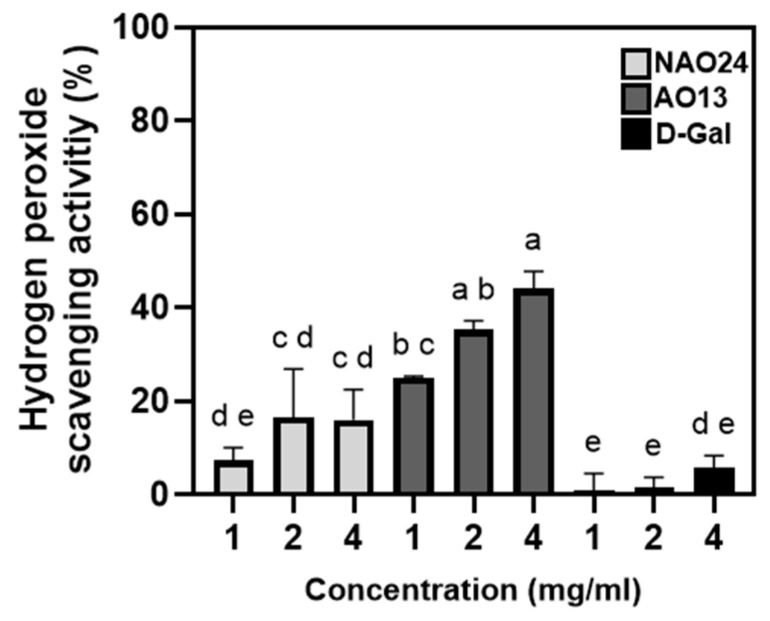
H_2_O_2_-scavenging activity of NAO24, AO13, and d-Gal. Different letters indicate significant differences (*p* < 0.05).

H_2_O_2_-scavenging assays revealed that NAO24 and d-Gal were deficient in antioxidant activity, with less than 20% H_2_O_2_-scavenging activity, whereas AO13 exhibited higher scavenging activity than NAO24 and d-Gal at all concentrations tested. AO13 showed scavenging activities of 25.0%, 35.3%, and 44.0% at 1, 2, and 4 mg/mL, respectively, indicating a concentration-dependent effect (Figure 6).

### 2.5. Cell Viability and Protective Effect of NAO24, AO13, and d-Gal against H_2_O_2_-Induced Oxidative Stress in HDFs

**Figure 7:**
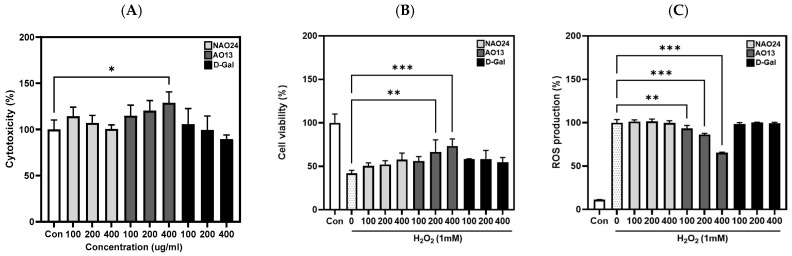
Protective effects of NAO24, AO13, and d-Gal against H_2_O_2_-induced oxidative stress in HDFs. (**A**) Cytotoxicity, (**B**) cell viability, and (**C**) ROS production. Values are mean ± SD (*n* = 3). * *p* < 0.05, ** *p* < 0.01, *** *p* < 0.001 compared with the H_2_O_2_-induced group.

Cytotoxicity assays of NAO24, AO13, and d-Gal using HDFs revealed cell viabilities of more than 90% at all concentrations tested, indicating that the mixtures were not cytotoxic. Additionally, NAO24 and d-Gal did not affect cell proliferation, whereas AO13 promoted cell proliferation in a dose-dependent manner (Figure 7A). The protective effects of NAO24, AO13, and d-Gal were investigated under H_2_O_2_-induced oxidative stress. When HDFs were treated with 1 mM H_2_O_2_, their viability decreased to 42.0% compared with that of the H_2_O_2_ non-treated control. Pretreatment with NAO24 and d-Gal at all concentrations tested before H_2_O_2_ exposure showed no significant improvement in cell viability. Notably, pretreatment with AO13 at 100, 200, and 400 μg/mL increased the cell viability to 56.0%, 66.6%, and 73.2%, indicating a dose-dependent effect (Figure 7B). H_2_O_2_ exposure increased intracellular ROS levels. Pretreatment with NAO24 and d-Gal did not affect the ROS levels, whereas treatment with AO13 decreased intracellular ROS levels to 93.4%, 86.4%, and 65.5%, indicating a dose-dependent effect (Figure 7C).

## 3. Discussion

Agarases can hydrolyze agarose into ENAOSs, EAOSs, ONAOSs, and OAOSs with different DPs. Although various agar oligosaccharide activities have been reported, most studies have focused on the activities of ENAOSs [3,6]. Hence, it is essential to analyze the physiological activities of EAOSs, ONAOSs, and OAOSs, as well as the agarases responsible for their production.

In this study, we identified and characterized α-NAOSH from *G. agarilyticus* JEA5. GH117 is similar to GH43 in that it has a five-bladed β-propeller fold structure, and the catalytic residues are conserved [18]. However, the conserved SxAxxR motif is a unique feature of the GH117 family, and we confirmed that Gaa117 has this unique motif. The SxAxxR motif, localized in the helix-turn-helix domain, is minimally required for the multimerization of GH117 family members. Swapping of the helix-turn-helix domain through the interaction of two monomers is responsible for dimerization [8,19]. The conservation of the SxAxxR motif and the amino acid residues involved in dimer formation (Arg-17, Glu-286, and Gln-291) suggest that Gaa117, like many other NAOSHs, is a dimer.

The optimum temperature for rGaa117 activity was 35 °C. This is comparable to that of most reported α-NAOSHs, the optimal temperature for which is approximately 30 °C [20,21,22,23,24]. Ahg786 from *Gayadomonas joobiniege* G7 and sdNABH from *Saccharophagus degradans* 2–40, exceptionally, show maximum activity at 15 °C and 42 °C, respectively [9,25]. Most reported α-NAOSHs exhibit maximum activity at pH 6.0–7.0 [20,22,23,25,26,27,28,29,30]. Similarly, rGaa117 showed optimum activity at pH 6.0 and maintained more than 60% of its activity in neutral conditions. The metal ions Mn^2+^, Ca^2+^, and Fe^2+^ increased rGaa117 activity. In previous studies, α-NAOSH activity was not affected by the presence of specific metal ions, but by the presence of various metal ions. For example, the activity levels of ScJC117 from *Streptomyces coelicolor* A3(2), WU-0601 from *Cellvibrio* sp., and MK03 from *Bacillus* sp. were enhanced in the presence of Mg^2+^, Ahg558 and Ahg786 from *Gayadomonas joobiniege* G7 in the presence of Mn^2+^, WU-0601 from *Cellvibrio* sp. and AhgI from *Gelidium amansii* in the presence of Ca^2+^ [20,21,24,27,28,29]. To our knowledge, this is the first study to report that Fe^2+^ enhances α-NAOSH activity. Previously, we identified and characterized extracellular β-agarases, Gaa16A and Gaa16B, which contain a signal peptide and produce NA4 and NA4 or NA2, respectively. Interestingly, the activity levels of Gaa16A and Gaa16B were also increased in the presence of Mn^2+^, Fe^2+^, and Ca^2+^ [31,32]. Many agar-degrading bacteria have diverse agarases to degrade agarose into monosaccharides for energy production [33]. In the agarolytic pathway of Gram-negative bacteria, agarose is first hydrolyzed into NA4 or NA2 by extracellular agarase because of the considerable molecular weight of agarose. The NA4 and NA2 are transported into the periplasm and further degraded by α-NAOSH into monosaccharides containing d-Gal and l-AHG [6]. Therefore, the increase in agarase activity in the presence of Mn^2+^, Fe^2+^, and Ca^2+^ suggests that these metal ions are crucial for the energy metabolic pathway to produce monosaccharides in *G. agarilyticus* JEA5. Kinetic parameter analysis revealed that the *K_cat_*/*K_m_* ratio of rGaa117, reflecting its catalytic efficiency, was 15 s^−1^/mM, which is notably higher than the value of 2.65 s^−1^/mM reported for BpGH117 [26].

The hydrolysis products of rGaa117 exhibited a pattern similar to those of other α-NAOSHs, indicating its ability to cleave the α-1,3-linkage at the non-reducing end of NAOSs [20,21,22,26,29]. However, rGaa117 exhibited relatively lower hydrolytic activity towards NA4 than NA2 and NA6. This difference in activity could be attributed to various factors related to enzyme–substrate interactions, such as accessibility of the active site and the proper orientation of substrates for binding [34,35]. Further analysis related to enzyme–substrate structure would facilitate better understanding of this difference in activity. Additionally, NAO24 (ENOAS mixture) and AO13 (OAOS mixture) were produced by combining rGaa117 and rGaa16B, and the antioxidant activities in terms of the structures of the agar oligosaccharides were compared. In the present study, AO13 demonstrated 2.75-fold higher H_2_O_2_-scavenging activity than NAO24 at a concentration of 4 mg/mL. The difference in activity is attributed to the structural differences between AOSs and NAOSs, with AOSs having d-Gal at the non-reducing end and NOASs having l-AHG at this position [13]. The antioxidant properties of agar oligosaccharides vary depending on their source material. Red algae, including *Gracilaia* and *Gelidium*, have complex cell walls that are primarily composed of agar but also contain additional polysaccharides such as cellulose, mannan, and xylan. Therefore, multiple purification processes are required to extract agar from red algae [36]. Our results are consistent with previous findings that NAOSs obtained from commercial agar showed no 1,1-diphenyl-2-picrylhydrazyl (DPPH) radical inhibition activity, whereas AOSs obtained from commercial agar showed high DPPH radical inhibition [13,37]. However, NAOSs obtained from raw *Gracilaia* or *Gelidium* extracts exhibited significant radical scavenging activity in both DPPH and 2,2′-azino-bis-3-ethylbenzthiazoline-6-sulphonic acid (ABTS) assays, which is potentially attributable to other constituents in the unrefined red algae hydrolysates [38,39].

ROS significantly contribute to skin aging by driving extracellular matrix degradation in the dermis, leading to the formation of wrinkles [40]. Therefore, H_2_O_2_-induced HDFs are commonly used as a model to investigate anti-skin-aging activity [41,42]. We found that AO13 exhibited no cytotoxicity in HDFs but promoted cell proliferation. In contrast to NAO24, AO13 provided significant protection in H_2_O_2_-induced HDFs by scavenging intracellular ROS. Therefore, AO13 has potential in the cosmetic and pharmaceutical industries owing to its ability to protect the skin from oxidative stress. Similarly, AOSs prepared through acid hydrolysis have demonstrated antioxidant activity by scavenging intracellular ROS in H_2_O_2_-induced hepatocytes [17]. Furthermore, AOSs enhanced the activity of antioxidant enzymes, including superoxide dismutase and glutathione peroxidase, in rats [17]. While the α-1,3 linkage of agarose is predominantly cleaved by acid to produce AOSs, this method has disadvantages in that it is difficult to obtain AOSs of specific DPs, and their structures are easily destroyed [4]. In contrast, enzymatic hydrolysis preserves the structure, can produce AOSs with desired DPs, is operationally simple, and is energy efficient because it proceeds at lower temperatures than acid hydrolysis [43]. Therefore, the effective production of OAOSs with antioxidant properties using rGaa117 offers possibilities for diverse applications.

## 4. Materials and Methods

### 4.1. Sequence Analysis and Cloning of Gaa117

The agar-degrading bacterium *G. agarilyticus* JEA5 was previously isolated from Jeju Island (Korea), and a cDNA database was established using next-generation sequencing [44]. The sequence of the predicted α-NAOSH was identified using NCBI BLAST, and the enzyme was termed Gaa117. The NCBI-CDS [45] and SMART online servers [46] were used to identify characteristic domains, and the SignalP 6.0 server [47] was used to predict signal peptides. Clustal Omega [48] and Color Align Conservation tools [49] were used to compare Gaa117 with its homologs. Genomic DNA from *G. agarilyticus* JEA5 (KCCM43129) was isolated and purified using an AccuPrep Genomic DNA Extraction Kit (Bioneer, Daejeon, Republic of Korea). The coding region of Gaa117 was amplified using Ex Taq (TaKaRa Bio, Shiga, Japan) and the forward primer 5′-GGAAGGATTTCAGAATTCATGTCAGATCAACACATTAAGCAAAAG-3′ (*Eco*RI site is underlined) and reverse primer 5′-CTGCAGGTCGACTCTAGATTATACGCCCGAGCCAC-3′ (*XBa*I site is underlined). The amplicon was purified using an AccuPrep PCR/Gel Purification Kit (Bioneer), ligated into pMal-c2x (pMal-c2x-Gaa117) using an Ez-Fusion Cloning Kit (Enzynomics, Daejeon, Republic of Korea), and transformed into competent *E. coli* DH5α cells. The plasmid was extracted from the cells using an AccuPrep Nano-Plus Plasmid Mini Extraction Kit (Bioneer) and sent to Macrogen (Seoul, Republic of Korea) for sequence confirmation. Finally, the plasmid was transformed into competent cell *E. coli* BL21 (DE3) cells for expression.

### 4.2. Overexpression and Purification of rGaa117

*E. coli* BL21 (DE3) cells harboring pMal-c2x-Gaa117 were cultured in Luria–Bertani medium supplemented with 100 mM glucose and 100 μg/mL ampicillin at 37 °C. When the bacteria reached the mid-logarithmic phase, 0.25 mM isopropyl β-d-1-thiogalactopyranoside (IPTG) was added and the bacteria were further cultured at 20 °C for 24 h. The cells were harvested by centrifugation at 1800× *g* for 20 min and resuspended in column buffer. The cells were stored at −20 °C overnight and then thawed on ice. Cell supernatant was obtained through sonication and centrifugation at 13,000× *g* for 30 min and soluble protein was purified using the pMal Protein Fusion and Purification system (New England Biolabs, Ipswich, MA, USA). Maltose in the purified protein fraction was removed using 50 kDa Amicon Ultra 15 Centrifugal Filters (Merck KGaA, Darmstadt, Germany). The rGaa117 purity and concentration were assessed using SDS-PAGE and a BCA Protein Assay Reagent Kit (Thermo Fisher Scientific, Waltham, MA, USA), respectively.

### 4.3. Enzyme Assay

The dinitrosalicylic acid method, which measures the amount of reducing sugars using d -Gal as a standard [50], was used to determine the activity of rGaa117. NA2, obtained by hydrolyzing agarose with β-agarase Aga50D, was used as a substrate [51]. A reaction mixture containing 2.5 μg/mL rGaa117 and 25 mM NA2 was prepared in a proper buffer and incubated at 35 °C for 10 min. The optimal temperature for rGaa117 was identified by testing temperatures ranging from 30 °C to 70 °C in 5 °C intervals. The optimal pH conditions were investigated using citrate phosphate buffer (pH 3.0–6.0), phosphate buffer (pH 6.0–8.0), and glycine–NaOH buffer (pH 8.0–10.0). To analyze thermostability, rGaa117 was subjected to pre-incubation at 30–40 °C for 0–120 min, after which the residual enzyme activity was determined. The effects of various metal ions on rGaa117 were investigated in a phosphate buffer (pH 6.0) containing MgCl_2_, MnCl_2_, NaCl, CaCl_2_, KCl, CuSO_4_, ZnSO_4_, FeSO_4_, or EDTA at a final concentration of 2.5 mM or 5 mM. The kinetic parameters of rGaa117 were measured using NA2 in the concentration range of 0.05–1.6 μmol, under optimal conditions. The *K_m_* and *V_max_* values were calculated using GraphPad Prism 10.3.0 (GraphPad Software, La Jolla, CA, USA).

### 4.4. TLC Analysis of the Hydrolyzation Products

TLC was used to analyze the hydrolyzation products of rGaa117. The reaction mixture included 12.5 μg rGaa117 and 50 μg NA2, A3, NA4, A5, NA6, or A7. Following incubation at 35 °C for 1 h, the reaction products were loaded onto silica gel 60 F_254_ (Merck KGaA) and developed using a solvent composed of *n*-butanol/ethanol/water (3:1:1). Spots were visualized using orcinol/H_2_SO_4_ reagent and incubation in a dry oven at 100 °C for 5 min. NA2, A3, NA4, A5, NA6, and A7 (Biosynth, Staad, Switzerland) were used as standards. d-AHG was used instead of l-AHG because the latter is difficult to obtain commercially (Dextra, Bangkok, Thailand).

### 4.5. Preparation of Mixtures of ENAOSs and OAOSs

One gram of agarose was completely dissolved in 100 mL of distilled water through heating. The agarose solution was incubated with the β-agarase Gaa16B at 50 °C overnight to completely hydrolyze the agarose into the ENAOSs composed of NA2 and NA4 [31]. NA2 and NA4 were further completely hydrolyzed through incubation with rGaa117 at 30 °C overnight, resulting in the production of OAOSs. The reaction mixture was incubated at 95 °C for 10 min to stop the enzyme activity. The reaction products were confirmed using TLC and freeze-dried for subsequent experiments. The ENAOSs produced by rGaa16B were designated NAO24, whereas the OAOSs produced by rGaa117 were designated AO13.

### 4.6. H_2_O_2_-Scavenging Activity Assay

The H_2_O_2_-scavenging activity of the enzyme was assessed using the Muller method [52]. First, 20 μL of NAO24, AO13, or d-Gal at different concentrations was mixed with 100 μL of 0.1 M phosphate buffer (pH 5.0) and 20 μL of 10 mM H_2_O_2_. The mixtures were incubated at 37 °C for 5 min. Then, 30 μL of 1.25 mM ABTS solution and 0.25 unit/mL peroxidase were added, and the mixtures were further incubated at 37 °C for 10 min. The absorbance of the solutions at 450 nm was measured and recorded using a Multiskan GO spectrophotometer (Thermo Fisher Scientific).

### 4.7. Cell Culture

HDFs (ATCC PCS-201-012, American Type Culture Collection, Manassas, VA, USA) were grown in DMEM/F12 (3:1) supplemented with 10% fetal bovine serum and 1% penicillin–streptomycin in a 5% CO_2_ environment at 37 °C.

### 4.8. Cell Viability Assay and ROS Production Measurement

To assess the effects of NAO24, AO13, and d-Gal on cell viability, HDFs were seeded in a 96-well plate at a density of 0.5 × 10^4^ cells/well. The cells were exposed to various concentrations of NAO24, AO13, and d-Gal for 24 h and then exposed to 1 mM H_2_O_2_ for 1 h. The medium was removed and the cells were incubated with 5 mg/mL 3-(4,5-dimethyl-2-thiazolyl)-2,5-diphenyl-2-H-tetrazolium bromide solution for 3 h. After the addition of dimethyl sulfoxide, the absorbance at 570 nm was measured. 2′,7′-Dichlorofluorescein diacetate (DCFH-DA) was used to measure intracellular ROS levels. HDFs were seeded and treated with the NAO24, AO13, d-Gal, and H_2_O_2_, as described above, and then incubated with 20 μM DCFH-DA for 30 min. Then, the fluorescence was measured using a fluorescence microplate reader at λ_ex_ = 485 nm and λ_em_ = 528 nm using a Synergy HT microplate reader (BioTek, Winooski, VT, USA) [53].

### 4.9. Statistical Analysis

Data are mean ± SD from triplicate experiments. The data were analyzed using a one-way ANOVA followed by Dunnett’s and Tukey’s multiple comparison tests using GraphPad Prism 10.3.0 (GraphPad Software, Inc., La Jolla, CA, USA). Statistical significance was set at *p* < 0.05.

## 5. Conclusions

α-NAOSH from *G. agarilyticus* JEA5 (Gaa117) was identified and overexpressed. Following purification, its enzymatic properties were characterized. rGaa117 showed the highest catalytic efficiency among known α-NAOSHs. When combined with β-agarase, rGaa117 generated OAOSs, which have higher H_2_O_2_-scavenging activity than ENAOSs. To our knowledge, this study was the first to investigate the effects of agar oligosaccharides on HDFs. OAOSs exhibited high ROS-scavenging activity in H_2_O_2_-induced HDFs, indicating that they may be applicable in cosmetics and pharmaceuticals for the prevention of skin aging. We only confirmed the activity of OAOSs composed of l-AHG and A3; future research needs to be conducted to produce EAOSs and investigate their activity.

## Data Availability

The original contributions presented in the study are included in the article/Supplementary Materials, further inquiries can be directed to the corresponding author.

## Supplementary Materials

The following supporting information can be downloaded at: https://www.mdpi.com/article/10.3390/md22110495/s1, Figure S1: Hydrogen peroxide (H_2_O_2_)-scavenging activity of d-galactose (d-Gal); Figure S2: Protective effect of d-Gal on H_2_O_2_-induced oxidative stress in human dermal fibroblasts.
