# Supplementary material for: Odd-Numbered Agaro-Oligosaccharides Produced by α-Neoagaro-Oligosaccharide Hydrolase Exert Antioxidant Activity in Human Dermal Fibroblasts
Source: Mar Drugs. 2024 Nov 3;22(11):495. doi: 10.3390/md22110495 (PMC11595537; doi:10.3390/md22110495)
Supplement: Supplementary file 1 [file marinedrugs-22-00495-s001.zip › marinedrugs-3268251-supplementary.pdf]

Supplementary materials

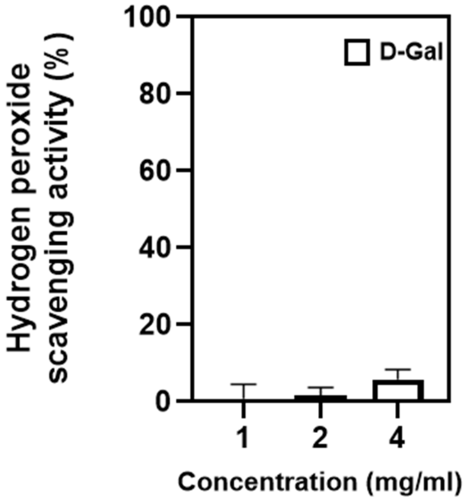

Figure S1. Hydrogen peroxide-scavenging activity of D-galactose (D-Gal).

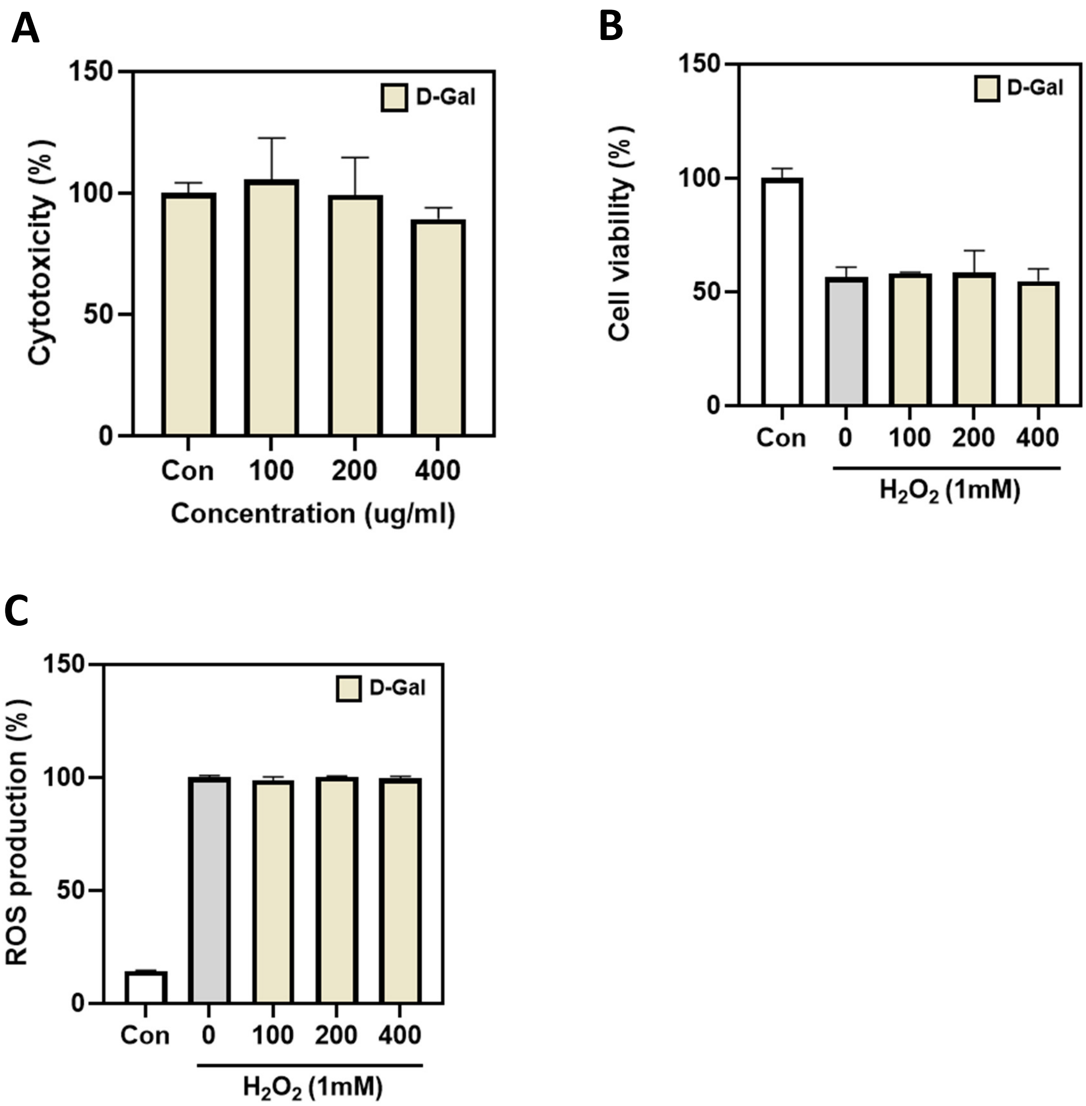

Figure S2. Protective effect of D-galactose on hydrogen peroxide-induced oxidative stress in human dermal fibroblasts. (A) Cytotoxicity, (B) cell viability, and (C) reactive oxygen species (ROS) production.
